# Interannual variability of leaf area index of an evergreen conifer stand was affected by carry-over effects from recent climate conditions

**DOI:** 10.1038/s41598-018-31672-3

**Published:** 2018-09-11

**Authors:** Akihiro Sumida, Tsutomu Watanabe, Tomiyasu Miyaura

**Affiliations:** 1 Institute of Low Temperature Science, Hokkaido University, N19W8, Sapporo, 060-0819 Japan; 2 Faculty of Science and Technology, Ryukoku University, Seta Oe-cho, Otsu, Shiga 520-2194 Japan

## Abstract

Despite the relevance of leaf area index (LAI) to forest productivity, few studies have focused on the interannual variability of LAI of an evergreen stand and its relationship with stand growth and meteorological factors. We estimated the change in LAI of an evergreen conifer (*Chamaecyparis obtusa*) stand over 19 years from a dataset using allometric methods. The LAI varied between 7.1 and 8.8 m^2^ m^−2^, with a 95% confidence interval of <1.1 m^2^ m^−2^ over the 19 years. This LAI range was maintained such that the gradual increase in leaf area (LA) of the largest trees counterbalanced the gradual loss in LA of the smallest trees. Meanwhile, more trees showed a temporary decrease in LA in years with low summer precipitation. The LAI and current-year mean temperature for July and August (*T*_JA_) were weakly correlated, whereas the correlation coefficient increased (*r* = 0.93) when LAI was correlated with the moving average *T*_JA_ over the previous 6 years, which agreed with the estimated turnover time of canopy foliage. The annual stem biomass growth rate was significantly positively correlated with summer precipitation, but not with LAI. These results will be useful for refining models in studies on forest growth and global climate change.

## Introduction

The leaf area index (LAI) of a forest is an important factor for estimating the primary productivity of the stand^1–5^. Hence, it is an important component for modelling and predicting the productivity of terrestrial ecosystems^6^. After a peak at relatively young stand ages, the LAI of a forest remains within a specific range^7–9^, although its value for older forests may be smaller or larger^10,11^ than that for younger mature stands.

Among the environmental factors that can affect the LAI of a stand, precipitation, ambient temperature, and water availability (as assessed e.g. by the ratio of potential evapotranspiration to precipitation) are the most well-studied^12–14^. Among stands in different regions where annual precipitation is low (<1500 mm), the LAI generally increases in line with annual precipitation^12,15,16^. The relative importance of precipitation may also depend on the soil water level^17^. In a biome with sufficient annual precipitation (>1500 mm), the LAI does not always increase with a further increase in annual precipitation, because it can also be limited by other factors^15,18^. For example, soil fertility affects the LAI in combination with air temperature and precipitation levels^19–22^. While the relationship between the LAI of a forest and meteorological factors is widely accepted, many of the abovementioned studies concluded that the details of this relationship are still obscure.

While the LAI of a closed deciduous stand is known to vary interannually to some extent^23,24^, it is unknown whether this is also true for evergreen stands, probably because of the lack of a reliable and convenient method to estimate the LAI of an evergreen stand over time^25^. Generally, the difference between current-year LAI and previous-year LAI of a stand is calculated by the difference between the amount of leaf area newly produced in the current year and the one lost between the previous and current years as e.g. leaf fall and grazing. In evergreen stands, estimating the former is very difficult. Previous studies have suggested that the interannual variability of LAI in evergreen stands relates to the interannual variability of the water balance of forests^26^, but the LAI estimates in such studies have depended on optical or remote-sensing based methods, which generate uncertainties in their estimates of LAI^25^. If the LAI of an evergreen stand does vary interannually, then the extent of this variability, the factors affecting it, and its effects on forest productivity are poorly understood. An among-stand comparison conducted by Smith *et al*.^27^ showed that net photosynthetic production did not strongly depend on LAI in evergreen conifer forests in which LAI had reached a certain level.

In contrast with environmental factors, internal factors such as the population dynamics of a stand have not been considered as possible factors affecting LAI. However, in an even-aged stand, the biomass increases with stand age and tree density decreases because of tree death from intraspecific competition (self-thinning)^28,29^. Within an even-aged stand, trees can have increasing or decreasing crown sizes^30,31^, suggesting that the competitive status of a tree affects the pattern of changes in its leaf area (hereafter ‘tree LA’) over time. Little is known about among-tree variation of the change in tree LA over time, or about the effects of the loss of tree LA due to the death of individual trees on the maintenance of stand LAI over time. In addition, the effects of meteorological factors on population dynamics have seldom been studied for evergreen stands through long-term observations of a given stand.

One of the most common direct methods for estimating LAI continuously over time is to combine non-destructive monitoring of tree dimensions (e.g. stem diameter and tree height) with an allometric method for estimating tree LA^25^. However, this method has rarely been used to study evergreen species^32,33^, probably because of the difficulty in estimating tree LA over time using plural among-tree allometric equations to fit different stand ages^8,25,33^. The allometric relationship deduced from pipe model theory^34,35^ has been reported to be insensitive to stand-age differences when estimating the amount of leaves for individual trees^8,36^. It proposes an allometric relationship between stem diameter at the crown base (*D*_CB_) and the amount of leaves on an individual tree (this method is hereafter termed ‘pipe model allometry’). Although care is needed because differences in site fertility may lead to differences in the coefficients of pipe model allometry among sites^35,37^, continuous measurements of *D*_CB_ can provide a non-destructive estimate of tree LA over time. The disadvantage of this method is that it requires laborious tree climbing to measure *D*_CB_.

In this study, we tested whether the mechanisms of LAI maintenance and its interannual variability could be identified by monitoring the changing patterns of tree LA with age. We estimated tree LA and stem dry weight (*W*_S_) for each living tree in each year from a dataset of intensive measurements^31^ using allometric equations for an evergreen conifer (hinoki cypress, *Chamaecyparis obtusa* (Siebold & Zucc.) Endl.) forest over a 20-year period. Possible errors of the tree LA values predicted using pipe model allometry were evaluated, and then an error propagation method^38^ was used to provide confidence intervals for LAI each year. This method was also applied when estimating the stand stem biomass (*B*_STEM_) from the *W*_S_ of trees. We then examined the contribution of changes in tree LA over time to the interannual variability of LAI. We also examined whether an increase in *B*_STEM_ for a given year (∆*B*_STEM_) was affected by the LAI of the same year, and evaluated the relationships between some meteorological factors and interannual variability in LAI and ∆*B*_STEM_. The meteorological factors were chosen through trial-and-error testing of monthly meteorological data recorded at the nearest weather station. Finally, we considered how evergreenness might be related to the maintenance of LAI of a particular tree species. The results of this study increase our understanding of the causes of interannual variability of LAI and its relationship with meteorological factors. Addressing these questions is important for improving projections of global change that use individual-based models incorporating plant physiological and ecological processes and forest–atmosphere interactions^6,39^.

## Results

### Chronological changes in tree leaf area in relation to crown dynamics

**Figure 1:**
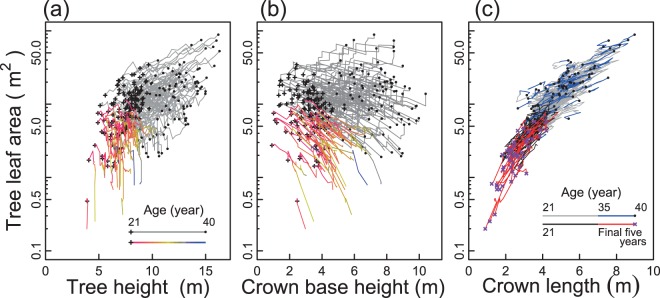
Time-trajectories of relationships between (**a**) tree leaf area (tree LA, m^2^) and tree height (m), (**b**) tree LA and crown base height (m), and (**c**) tree LA and crown length (=tree height − crown base height, m) of individual trees. In (**a**,**b**), data points for initial year (21-years stand age) and final year (40-years stand age) are indicated by ‘+’ symbols and closed black circles, respectively. Coloured curves without black circles show dying trees (i.e. 50 trees that died before 40 years of stand age). For dying trees, changes in colour of curved line indicate changes in stand age. Trees that survived during whole observation period (92 trees in total) are indicated with grey curved lines. In (**c**), grey lines with blue lines represent surviving trees. Blue lines show the trajectory from 35 to 40 years of age. Black lines with red lines indicate dying trees, where the red lines indicate the final five years before their death (two trees that died because of breakage in upper stem are not shown).

Whereas tree heights generally increased with age, the tree LA of some trees decreased from their initial values (Fig. 1a). In particular, the 50 trees that died before 40 years (hereafter ‘dying trees’) showed a continual decrease in tree LA over the years before their death. This decrease in tree LA was accompanied by reduction in crown length (Fig. 1c); crown length reduction occurs when the growth rate of tree height is smaller than the rate of the rise of crown base that results from the death of the lowest living branches in the crown. Given the tapered shape of the tree stem within the crown, a rapid rise of the crown base can lead to a decrease in the crown base stem diameter (*D*_CB_), which is used to estimate the tree LA by pipe model allometry. Thus, Fig. 1 illustrates that the decrease in tree LA was accompanied by the reduction in crown length due to progressive death of the lowest living branches in the crown. Figure 1 also illustrates that the stand consisted of trees with increasing LAs and those with decreasing LAs with age, and that trees showing an overall trend for increasing tree LA occasionally showed a reduced tree LA in some years.

### Contribution of individual trees to LAI and *B*_STEM_ of the stand

**Figure 2:**
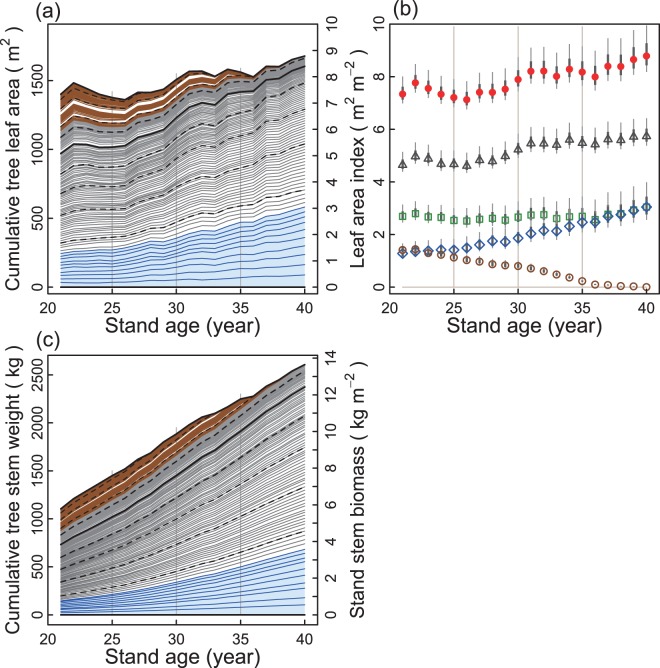
Changes with age in tree leaf area (tree LA) and stem weight (*W*_S_) of each tree as related to changes with age in stand LAI and stand stem biomass (*B*_STEM_). In (**a**,**c**), each curved line (individual tree) shows changes in tree LA or *W*_S_ with stand age. Lines are ordered from bottom up, starting with tree with largest LA at 40 years of age. Hence the uppermost line in (**a**) shows changes in the sum of the tree LAs of all living trees with age, which correspond to the changes in LAI in (**b**). Brown curved lines in upper section indicate dying trees; lines are ranked from bottom to top in order of later age of death. The thickness of brown bundle in this upper section in (**a**,**c**) corresponds to the sum of the tree LAs and that of the stem weights, respectively, for the dying trees. Grey curved lines in the middle and the blue curved lines in the bottom section in (**a**,**c**) are medium trees and vigorous trees, respectively. The 142 trees measured in initial year were divided into 10th percentiles of number of trees and separated by broken lines. Thus, a bundle of curved lines enclosed by two broken lines includes 14 or 15 trees in initial year. Thick curved line indicates 50th percentile. In (**b**), changes with age in stand LAI (closed circles), relative contribution to stand LAI of dying trees (open circles), vigorous trees (open diamonds), and medium trees (open triangles), and sum of LAs of vigorous and dying trees (open squares) are indicated. Thick and thin vertical bars indicate defined error bars approximating 1 × s.d. and 2 × s.d., respectively.

The presence of trees that showed either increasing or decreasing tree LA contributed to maintenance of stand LAI within a certain range. The LAI varied between 7.1 (at 27 years) and 8.8 m^2^ m^−2^ (at 40 years), and the 95% confidence intervals above and below each LAI estimate were <1.12 and <0.58 m^2^ m^−2^, respectively (both at 40 years) (Fig. 2a,b). The LAI also exhibited relatively long (ca. 5−10 year) fluctuation cycles over the 19 years of data collection (Fig. 2a,b).

Hereafter the 142 living trees initially measured are grouped into ‘vigorous’, ‘medium’, and ‘dying’ for convenience. The vigorous trees are the 10 trees that retained the largest tree LAs in the initial year, and the medium trees are the 82 trees between the vigorous and dying trees in the initial year. Of the three tree groups, the vigorous and dying trees had similar sums for tree LA in the initial year (~244 and ~269 m^2^, respectively), contributing ~1.3 m^2^ m^−2^ and ~1.4 m^2^ m^−2^, respectively, to the stand LAI in the initial year (Fig. 2b). Thereafter, these two groups showed contrasting changes in their total LAs over time. The sum of the LA of the vigorous trees increased with stand age and reached 3.0 m^2^ m^−2^ at 40 years of stand age, whereas that of the dying trees decreased and reached zero before 40 years (Fig. 2a,b). This implied that there were drastic changes in the respective proportions of LAI contributed by the vigorous and dying trees. As a result, the combined sum of the tree LA of the vigorous and dying trees remained almost stable over the study period.

The growth curve of *B*_STEM_, which is indicated by the uppermost curved line in Fig. 2c, illustrated that the *B*_STEM_ of the stand increased by more than two-fold between 21 and 40 years of stand age. The vigorous trees tended to show roughly exponential increases in stem weights over the study period. However, the sum of the stem weights for the dying trees (indicated by the thickness of brown bundle in the upper section in Fig. 2c) peaked at 24 years of stand age, and then continuously decreased. This decrease was because their stem weights were excluded from the stand *B*_STEM_ as these individuals died. However, this decrease was so small that the total *B*_STEM_ of the stand increased continuously with only slight fluctuations.

### Climatic factors affecting interannual variability of LAI

**Figure 3:**
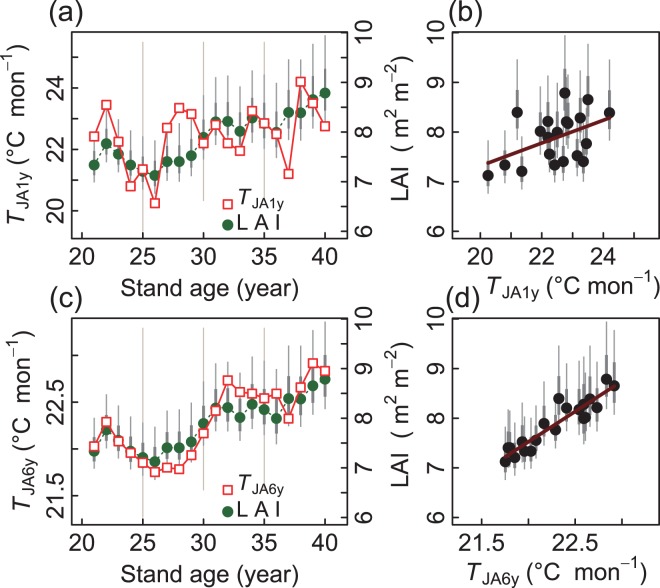
Relationships between summer temperatures (*T*_JA1y_, *T*_JA6y_) and LAI, where *T*_JA1y_ and *T*_JA6y_ correspond to current-year value and 6-year moving average, respectively. Left panels (**a**,**c**) show changes with stand age, in which LAI values are indicated by the vertical axis at right-hand side of each panel. Right panels (**b**,**d**) show regressions between corresponding variables in left-hand panels. See Table 1 for regression results. Thick and thin vertical bars indicate defined error bars approximating 1 × s.d. and 2 × s.d., respectively.

Next, we examined which climatic factors affected the interannual variability of LAI. Among the various combinations of monthly meteorological data examined, the average monthly mean air temperatures of July and August of each year (*T*_JA1y_; hereafter ‘summer temperature’) had a significant positive relationship with LAI (Fig. 3ab), although the relationship was not very strong (*R*^2^ = 0.159, *P* = 0.046; see Table 1). We did not find any other meteorological factors that were significantly related to the interannual variability of LAI. We also examined the effects of the precipitation in a single month on LAI, but did not find any significant relationships.

**Table 1:** Coefficients of regression analyses. See main text for abbreviations and units.

Figure number	Response variable	Predictor variable	Intercept	Slope	*R* ^2^	*F*	*P*	*df*
3b	LAI	*T*_JA1y_	2.73	**0.23**	0.159	4.59	0.046	1, 18
3d	LAI	*T*_JA6y_	−19.36	**1.22**	0.850	108	0.000	1, 18
—	LAI	*P*_July_	7.54	0.001	0.039	1.72	0.207	1, 17
—	LAI	*P*_MJJ_	7.99	−0.000	0.000	0.238	0.879	1, 17
4a	∆LAI	*R*_(∆LA<0)_	0.76	**−1.77**	0.738	51.6	0.000	1, 17
—	∆LAI	*P*_July_	−0.17	0.001	0.143	4.00	0.062	1, 17
—	∆LAI	*P*_MJJ_	−0.30	0.001	0.094	2.87	0.108	1, 17
—	∆LAI	*P*_July_/*E*_July_	−0.09	0.055	0.102	2.81	0.115	1, 15
4b	*R*_(∆LA<0)_	*P*_July_	0.532	**−0.00062**	0.231	6.39	0.022	1, 17
—	*R*_(∆LA<0)_	*E*_July_	0.219	0.0012	0.023	1.37	0.260	1, 15
4c	*R*_(∆LA<0)_	*P*_July_/*E*_July_	0.508	**−0.0412**	0.260	6.61	0.021	1, 15
4d	∆LAI	∆*B*_STEM_	−0.452	**1.276**	0.347	10.6	0.005	1, 17
5a	∆*B*_STEM_	LAI	0.578	−0.021	0.000	0.15	0.704	1, 17
5c	∆*B*_STEM_	*P*_MJJ_	0.111	**0.00046**	0.366	11.4	0.004	1, 17
—	∆*B*_STEM_	*P*_July_	0.321	0.00040	0.063	2.21	0.155	1, 17
—	∆*B*_STEM_	*P*_July_/*E*_July_	0.338	0.0228	0.036	1.60	0.224	1, 15

Slope values in boldface are significant at *P *< 0.05.

The estimated turnover time of the leaves in this stand was 4.3–6.3 years (see Materials and Methods). Therefore, we expected that if LAI was correlated with the summer temperature, the summer temperatures of not only the current year but those of the past several years when the leaves in the current-year canopy accumulated would affect the LAI of the current year. Hence, we calculated the moving averages of the summer temperatures of current and past *N*_Y_ years (*N*_Y_ = 1, 2, …, 10, where *N*_Y_ = 1 is the current year), and tried to find the number of years for calculating the moving average that generated the strongest correlation between the moving average and the current-year LAI. The correlation was highest when *N*_Y_ = 6, that is, when the moving average of the summer temperatures was taken for the current year and the previous 5 years (*T*_JA6y_) (*R*^2^ = 0.850, *P* = 0.000, Table 1 and Fig. 3d). The pattern of interannual fluctuations in LAI agreed well with *T*_JA6y_ (Fig. 3c), and the *R*^2^ values became smaller when *N*_Y_ was greater or less than 6 years (see Supplementary Information S4).

### Crown base rise as a morphological factor affecting ΔLAI

**Figure 4:**
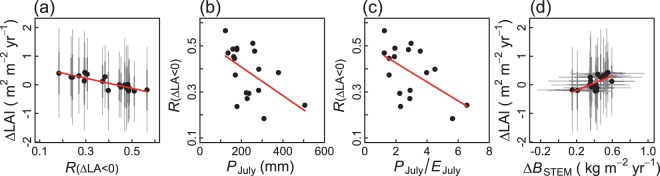
Relationships between (**a**) ratio of number of trees with negative ΔLAs (*R*_(ΔLA<0)_) and ΔLAI (*P* = 0.000). (**b**) July precipitation (*P*_July_) and *R*_(ΔLA<0)_ (*P* = 0.022), (**c**) ratio of *P*_July_ to July potential evaporation (*P*_July_/*E*_July_) and *R*_(ΔLA<0)_ (*P* = 0.021), and (**d**) stem biomass increment (Δ*B*_STEM_) and ΔLAI (*P* = 0.005). Thick and thin vertical bars in (**a**,**d**) indicate defined error bars approximating 1 × s.d. and 2 × s.d., respectively. See Table 1 for regression results.

The interannual variability of LAI was further investigated from another viewpoint. As shown in Fig. 1b,c, a decrease in tree LA was associated with an increase in the crown base height. If many trees had a decrease in tree LA in a year, then the LAI could decrease in the following year. To confirm this connection between the interannual variability of LAI and tree LA, the ratio of the number of trees with negative ΔLAs (ΔLA; tree LA of a year minus that of the previous year) to the total number of living trees (*R*_(ΔLA<0)_) was calculated for each year, and this ratio was compared with the rates of change in LAI (ΔLAI). As shown in Fig. 4a, ΔLAI had a negative relationship with *R*_(ΔLA<0)_. The value of *R*_(ΔLA<0)_ ranged from 0.2 and 0.6 over the 19 years, indicating that the ratio differed three times between the smallest and largest values in the 19 years, and the ΔLAI values were negative in nine of the 19 years (Fig. 4a). Further, we found that *R*_(ΔLA<0)_ was negatively related to both July precipitation (*P*_July_; *R*^2^ = 0.231, *P* = 0.022; Table 1) and the ratio of *P*_July_ to July potential evaporation (*P*_July_/*E*_July_; *R*^2^ = 0.260, *P* = 0.021; Table 1) (Fig. 4b,c). These results suggested that in years of low precipitation in July, more trees had branches dying in the lowest part of the crown. This led to an increase in the height of the crown base and a negative ΔLA, resulting in negative ΔLAI. Although the relationship between ΔLAI and *R*_(ΔLA<0)_ was significant (Fig. 4a), that between ΔLAI and *P*_July_ was less significant (*R*^2^ = 0.143, *P* = 0.062; Table 1), and we did not find other meteorological factors related to ΔLAI. In addition, *E*_July_ alone did not have a significant relationship with *R*_(ΔLA<0)_ (*R*^2^ = 0.023, *P* = 0.260; Table 1), nor did other monthly potential evaporation values (data not shown). *P*_July_ did not have a significant relationship with LAI, either (*R*^2^ = 0.039, *P* = 0.207; Table 1).

We then tested if ΔLAI showed any correspondence with the change in *B*_STEM_ (Δ*B*_STEM_) (Fig. 4d). The relationship between ΔLAI and Δ*B*_STEM_ was positive (*R*^2^ = 0.347, *P* = 0.005; Table 1), indicating that Δ*B*_STEM_ tended to be small in years with negative ΔLAI (Fig. 4d).

### Meteorological factors affecting interannual variability of the change in *B*_STEM_

**Figure 5:**
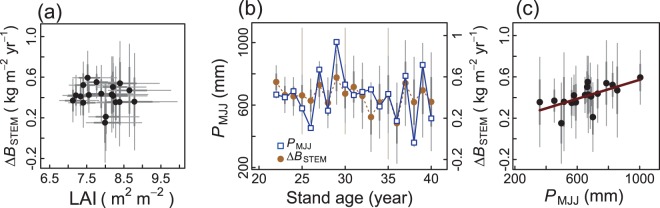
Factors affecting stand stem biomass increment (Δ*B*_STEM_). (**a**) Relationship between LAI and change in Δ*B*_STEM_, (**b**) changes in summer precipitation (*P*_MJJ_) and Δ*B*_STEM_ with stand age, and (**c**) relationship between *P*_MJJ_ and Δ*B*_STEM_. In (**b**), values of Δ*B*_STEM_ are indicated by the vertical axis on right-hand side of panel. See Table 1 for regression results. Thick and thin vertical bars indicate defined error bars approximating 1 × s.d. and 2 × s.d., respectively.

We had expected that years with relatively high LAI values would have large positive values of Δ*B*_STEM_. However, as shown in Fig. 5a, the relationship was not significant (*R*^2^ = 0.000, *P* = 0.704; Table 1). Thus, a year with high LAI did not always have a large increase in *B*_STEM_. We examined which climatic factors affected the interannual variability of Δ*B*_STEM_, and found that Δ*B*_STEM_ was positively related to the sum of precipitation for May, June, and July, *P*_MJJ_, (hereafter ‘early summer precipitation’) (*R*^2^ = 0.366, *P* = 0.004; Table 1 and Fig. 5b,c). Δ*B*_STEM_ was not significantly related to monthly precipitation of any of the three months alone (*P* > 0.05, data not shown), including *P*_July_ (Table 1). This early summer precipitation (*P*_MJJ_) was not significantly related to LAI and ΔLAI (*P* = 0.879 and 0.108, respectively; Table 1), suggesting that the major meteorological factors affecting Δ*B*_STEM_ differed from those affecting LAI and ΔLAI. The relationships between potential evaporation each month and Δ*B*_STEM_ and ΔLAI were also not significant (*P* > 0.05, data not shown).

## Discussion

### Individual-based interpretation of interannual changes in LAI: importance of dying trees

While the stand LAI varied between 7.1 and 8.8 m^2^ m^−2^, there was a drastic change over time in relative contributions of the tree groups of different statuses (i.e., vigorous *vs*. dying trees) to stand LAI (Fig. 2b). This finding suggested an individual-based mechanism whereby the LAI of a stand is maintained within a certain range despite the growth and death of component trees. In terms of competition among individuals, this process can be likened to the ‘LA haves’ (trees with the highest initial LAs) taking over the share of LA allotted to the ‘LA have-nots’ (those with the lowest initial LAs). The vigorous trees would naturally take over the LA share from the dying trees rather than from the medium trees, as the dying trees would be more susceptible to attack.

A plausible explanation for this phenomenon is that vigorous and dying trees tended to neighbour each other in the initial year. The forest canopy probably developed in such a manner that the crowns of the taller trees slowly took over space, thus preventing the crowns of the shorter trees from spreading, as is often the case with competition among neighbouring trees^40^. These spatial factors have not yet been thoroughly analysed. Further studies incorporating spatial pattern analyses are needed to test the above hypothesis.

From the viewpoint of interannual variability of LAI, the observation that dying trees were able to survive for many years even as their LA was decreasing (Fig. 1) would be important for LAI to be maintained within a certain range. If, instead, suppressed trees died abruptly after a short period of suppression, it would result in the sudden loss of their LA. This may have led to a substantial reduction in LAI. In addition, there were trees that showed a temporary decrease in LA among the trees that survived for the whole observation period (Figs 1 and 2). The sum of LAs for the trees with ΔLA < 0 accounted for between 9.9% and 42.8% of the stand LAI among years (see Supplementary Information S5). Even trees in the tallest height class showed a temporary decrease in tree LAs when the proportion of trees with ΔLA < 0 was high (see Supplementary Information S5). This suggests that the effect of *P*_July_ on *R*_(ΔLA<0)_ (Fig. 4) was exerted not only on suppressed trees but also on dominant trees in the stand.

Despite the death of dying trees, *B*_STEM_ never decreased during the observation period (Fig. 2c). However, the total basal area (sum of the stem cross-sectional areas at 1.3 m height of living trees) decreased from years 32 (44.22 m^2^ ha^−1^) to 33 (43.91), and from years 35 (44.97) to 36 (44.34) of stand age (see Table S1 of Sumida *et al*.^31^). This contrast between *B*_STEM_ and the total basal area is explained by the fact that in suppressed *C. obtusa* trees with almost no radial growth in the stem section below the crown, the portion of the stem within the crown maintained a certain level of radial growth, as we observed previously at the current study site^31^. This implies that if DBH only is used as a surrogate of the responses of biomass increase or productivity to climatic factors, then the effects of influential climatic factors may not be able to be detected. For example, if Δ*B*_STEM_ in the Δ*B*_STEM_‒*P*_MJJ_ relationship (Fig. 5c) was replaced by the annual change in total basal area at 1.3-m height (ΔBA, m^2^ ha^−1^), the significance of the relationship was lost (ΔBA = −0.372 + 0.0187 × *P*_MJJ_; *P* = 0.056, *R*^2^ = 0.152, *F* = 4.214 on 1 and 17 *df*). The use of detailed individual-based data from the Hinoki Data, which did not depend on conventional DBH and tree height for estimation of tree LA and *W*_S_, probably contributed to the successful detection of several significant effects of meteorological factors.

### Meteorological factors and interannual variability of LAI

The results shown in Fig. 3 supported our working hypothesis that the number of years for calculating the moving average of summer temperatures was approximately the same as the average leaf turnover time of the evergreen canopy. The high *R*^2^ value (=0.850) indicated that 85% of the variation of LAI in that relationship was explained by the summer temperatures of the past years during which the leaves in the current-year canopy accumulated. This ‘carry-over’ effect from past years’ meteorological conditions suggests that the LAI in a given year does not strongly respond to current-year meteorological conditions in an evergreen stand. It also highlights the importance of considering carry-over effects of meteorological conditions on the current conditions of trees.

The reason for the positive relationship between the summer temperature and LAI (Fig. 3d) is still unknown. One possible reason would be that, in terms of shoot-growth phenology, the summer temperatures that we used (July and August) represented the most favourable season for increasing the tree LA of *C. obtusa*. In central Japan, the growth in tree height of *C. obtusa* (i.e., the extension of the main trunk) generally starts in May and continues until October^41^. The extension of new current-year shoots occurs at the apices of the main annual shoots of the previous year on twigs, with scale leaves and subsequent lateral shoots being produced continuously during the growing season^42^. The cessation of shoot extension occurs later in *C. obtusa* than in other conifer genera such as *Picea*, *Pinus*, and *Larix*^43^, in which shoot extension growth generally ends by August. This difference in shoot extension phenology could be ascribed to the indeterminate shoot growth pattern of *C. obtusa*. It is therefore possible that a warmer growing period (i.e., warm July and August temperatures) contributed to greater leaf production and increased leaf accumulation in the canopy.

Other studies on LAI have highlighted the importance of water availability (see Introduction), but we did not find any significant relationships between LAI and precipitation or potential evaporation. This inconsistency with the results of previous studies may be because, as indicated by Gower^15^, rainfall did not strongly influence LAI at our study site, as the stand was located in a region where the climate is relatively cool and the average annual precipitation is sufficient (the minimum annual rainfall during the observation period was 1231 mm at 28 years of stand age). Although the positive relationship between ΔLAI and *P*_July_ was not significant (*P* = 0.062; Table 1), it seems plausible that less precipitation in summer could trigger the death of branches in the lower canopy and decrease LAI. Taking into account the positive relationship between the LAI and summer temperature (Fig. 3), the increase in LAI resulting from shoot growth and the decrease in the LAI resulting from the death of lower branches may be governed by different meteorological factors. That is, warm summer temperatures would increase the LAI, and less summer precipitation would decrease the LAI. Further research is needed to test this idea. It is also important to consider the biological characteristics of trees, such as shoot morphology, phenology, and physiology, to fully understand the retention of evergreen leaves in the canopy.

### Does stem biomass increase in a LAI-dependent manner?

The present study indicated that Δ*B*_STEM_ did not show a significant relationship with LAI (Fig. 5a). If LAI does not determine biomass growth, why did the trees accumulate more foliage than necessary for growth? Two explanations are possible on the basis of findings of previous studies. The first possibility is that, even if photosynthetic production was proportional to LAI, a substantial proportion of the carbohydrates by photosynthesis may have been used for purposes other than stem biomass production. A body of evidence suggests that carbon is not likely limiting to tree growth^18,44–46^; non-structural carbohydrates (NSC) are generally abundant within tree tissues, even in trees suffering environmental stresses, as NSC are essential for maintaining hydraulic functionality or osmotic adjustment^18,44,45,47^. A modelling study in which the amount of carbon used for woody biomass production was assumed to be supplied from the ‘remainder’ of the NSC allotted to physiological maintenance accurately reproduced actual woody biomass production^48^. If this is the case, stem biomass growth is not always proportional to canopy photosynthetic production, even if the photosynthetic production was proportional to LAI, resulting in the absence of a significant LAI–Δ*B*_STEM_ relationship. In addition, previous authors have pointed out that, although growth is mainly fuelled by the photosynthates of the current year^44,46^, drought is known to reduce stem growth before it affects photosynthetic production by decreasing cell expansion and differentiation for stem growth^18,45^. This explains why Δ*B*_STEM_ showed a significant positive relationship with early summer precipitation (Fig. 5c); that is, less early summer precipitation limited Δ*B*_STEM_ by negatively affecting cell expansion and stem growth.

The second possibility is associated with the growing evidence that the function of the evergreen canopy is not only photosynthetic production, but also nutrient storage, especially nitrogen^49,50^. Previous studies^44,51–53^ have suggested that a crucial enzyme in photosynthesis, RuBisCO, plays a role in nitrogen storage in evergreen species during winter, and the nitrogen stored in RuBisCO is remobilised to newly developing shoots in the following spring. Thus, the absence of a significant LAI–Δ*B*_STEM_ relationship may have reflected the role of evergreen canopy as storage, resulting in the LAI level exceeding that necessary for biomass growth. Both of the abovementioned possibilities likely partly explain the reason of the absence of a significant Δ*B*_STEM_–LAI relationship in the evergreen canopy. The significant positive relationship between ΔLAI and Δ*B*_STEM_ (Fig. 4d) suggests that Δ*B*_STEM_ synchronises with the amount of new leaves produced. This synchronisation is likely reasonable if trees need to secure the amount of conductive tissues that balances with the amount of new leaves produced^32^.

## Conclusions

To our knowledge, this is the first study to demonstrate quantitatively the maintenance of LAI of evergreen conifer species within a certain range based on chronological changes in the LA of individual trees. The LAI of *C. obtusa* was strongly affected by the carry-over effects of meteorological conditions of the preceding years when the current-year canopy leaves accumulated. This finding suggests that, in other studies, unknown carry-over effects may obscure the relationship between current-year biological conditions and current-year meteorology. The absence of a significant relationship between LAI and Δ*B*_STEM_ suggests that the view that the magnitude of LAI proportionally determines biomass production should be revisited. Early summer precipitation was observed to be the major factor affecting Δ*B*_STEM_. The present results will be useful for refining models in studies on forest growth and global climate change.

## Materials and Methods

### Study site and field measurements

In this study, we used a published, long-term stand observation dataset (hereafter the ‘Hinoki Data’), which is available in the online material for Sumida *et al*.^31^. The stand was an even-aged plantation of hinoki cypress (*Chamaecyparis obtusa* (Siebold & Zucc.) Endl.) located in the Experimental Forest of Nagoya University in Inabu, Aichi Prefecture, Japan. The area of the plot was 191 m^2^, and it was located ~970 m above sea level (a.s.l.) (35°12′14″N, 137°33′58″E) on a steep slope (average gradient 37°). The canopy of evergreen species is composed of leaves of several different cohorts. Therefore, it was important to know the mean turnover time of leaves as it can affect LAI. Miyaura^54^ investigated the relationships between tree size and leaf mass per tree, and determined the annual per tree leaf-litterfall rates by enclosing the crowns of several *C. obtusa* trees in a stand. Based on this investigation, the mean turnover time of the leaves of individual trees in the stand was estimated to be 4.3–6.3 years^54^.

The Hinoki Data is an annual record of non-destructive measurements of all living trees from the initial year (1977, 21-years stand age) to the final year (1996, 40-years stand age). Hence, it consists of 20 annual data sets observed over 19 years. The number of living trees decreased from 142 (~7400 ha^−1^) to 92 (~4816 ha^−1^) during the observation period as a result of self-thinning. One characteristic feature of the Hinoki Data is that stem girth measurements at 1-m intervals as well as tree height and crown base height (defined as the height just below the base of the lowest living branch) were measured by climbing up to the top of all living trees each year. Using these data, therefore, the stem volume of a tree can be directly calculated as the sum of the volumes of the 1-m stem segments for each tree and each year, without the need for allometric calculations. The other non-destructive measurements included the stem girth at the crown base, which was measured annually for each living tree. With these data, it is possible to estimate the LA of each tree in each year by applying pipe model allometry^34,35^. The LAs of the dying trees were also estimated when they were alive. The death of an individual tree was judged when the tree was climbed, by observing whether all living leaves were etiolated or had been lost. Further details of the measurements are available in Sumida *et al*.^31^.

### Estimation of LAI and *B*_STEM_ using allometric relationships for tree LA and *W*_S_

To estimate the tree LA and stem dry weight *W*_S_ for each tree in each year, the following allometric equations were deduced from data obtained by destructive sampling of *C. obtusa* trees. These data are available in Hagihara *et al*.^55^ (hereafter termed the ‘HYO Data’). The destructive sampling was carried out at four sites in central Japan, including one stand surrounding our study plot where the Hinoki Data were collected.

Although several allometric relationships were given in Hagihara *et al*.^55^, the relationship between *D*_CB_ and tree LA was not presented, and errors associated with log-transformation were not considered. To correct the bias associated with log-transformation^56,57^, a correction factor, CF, was calculated. Thus, the allometric equation for predicting tree LA (m^2^) from the records of *D*_CB_ (cm) in the Hinoki Data was as follows:

1 $${\rm{LA}}={{\rm{CF}}}_{{\rm{LA}}}\times 0.105\times {D}_{{\rm{CB}}}^{2.46},$$

where the correction factor CF_LA_ was 1.051. The scatterplot for this regression line is shown in Supplementary Information S1, with 95% prediction intervals (PIs) around the regression line^58^. See also S1-1 in Supplementary Information S1 for details of how equation (1) was derived. The LA of each tree in each year was estimated by using the ‘GB’ value (i.e. the abbreviation of stem girth [cm] at the crown base each year) from the Hinoki Data in equation (1) after converting the girth to diameter *D*_CB_. The LAI was calculated as the sum of tree LA each year divided by plot area.

The definition of LAI differs among papers^11,19^. In this study, the tree LA data used to derive equation (1) corresponded to half of the leaf surface area^55^. Hence, the tree LA defined in this way was used to estimate the LAI.

The second allometric equation derived from the HYO Data was the relationship between stem volume *V*_S_ (m^3^) and stem dry weight *W*_S_ (kg). Using the same procedure as that used to derive (1), we obtained the following equation:

2 $${W}_{{\rm{S}}}={{\rm{CF}}}_{{\rm{WS}}}\times 367.0\times {V}_{S}^{0.975},$$

where the correction factor CF_WS_ was 1.002. The scatterplot of the data with equation (2) is shown in S1–5 in Supplementary Information S1, with 95% PIs around the regression line. The same field method was used to obtain *V*_S_ in the collection of the HYO Data and the Hinoki Data (see S1–5 Supplementary Information S1). Equation (2) was used to estimate *W*_S_ from the annual records of *V*_S_ for each tree in the Hinoki Data. The *B*_STEM_ (kg m^−2^) for a given year was calculated by dividing the sum of stem mass of living trees by the plot area.

The rates of change in LAI (ΔLAI, m^2^ m^−2^ year^−1^) and *B*_STEM_ (Δ*B*_STEM_, kg m^−2^ year^−1^) for a given year *t* were calculated by subtracting the value at year *t* from that at year (*t* − 1). As the fieldwork for the Hinoki Data was conducted in autumn (after the growing season) each year, values of ΔLAI and Δ*B*_STEM_ in year *t* practically corresponded to changes that occurred during the growing season of that year.

### Propagation of errors originating from allometric methods for LAI, ΔLAI, and Δ*B*_STEM_

In this study, the errors of tree LA and *W*s estimated from the allometric equations were defined as the difference between the value estimated by an allometric equation and its 95% PIs. Here, we note that the PIs were calculated for natural-log-transformed values of LA and *W*s. The errors of natural-log-transformed regressions for equations (1) and (2) were normally distributed. However, when these logarithmic values were transformed into non-log values, the ranges of error distribution and the prediction intervals were greater in the region above the regression line than in the region below it (see S1-1 in Supplementary Information S1). Taking this into account, we first defined the error of an estimate of individual tree LA by separating it into the upper and lower regions of the estimate, as follows:

3a $${\delta }_{\mathrm{Upper}({{D}_{{\rm{CB}}}}^{\ast })}={{\rm{PI}}}_{\mathrm{Upper}({{D}_{{\rm{CB}}}}^{\ast })}\,-\,{{\rm{LA}}}_{({{D}_{{\rm{CB}}}}^{\ast })}$$

3b $${{\delta }}_{{\rm{Lower}}({{D}_{{\rm{CB}}}}^{\ast })}={{\rm{LA}}}_{({{D}_{{\rm{CB}}}}^{\ast })}\,-\,{{\rm{PI}}}_{\mathrm{Lower}({{D}_{{\rm{CB}}}}^{\ast })},$$

where ${{\rm{\delta }}}_{\mathrm{Upper}({{D}_{{\rm{CB}}}}^{\ast })}$ and ${{\rm{\delta }}}_{\mathrm{Lower}({{D}_{{\rm{CB}}}}^{\ast })}$ are the absolute values of the theoretical upper and lower limits, respectively, of the 95% PIs expressed as non-log values of LA. The calculations for ${{\rm{\delta }}}_{\mathrm{Upper}({{D}_{{\rm{CB}}}}^{\ast })}$ and ${{\rm{\delta }}}_{\mathrm{Lower}({{D}_{{\rm{CB}}}}^{\ast })}$ are given in S1–2 in Supplementary Information S1.

These errors for individual estimates of LA and *W*s were used to calculate the errors in the estimates of LAI, ΔLAI, and Δ*B*_STEM_ using error propagation rules^38^. For example, the error bounds for estimating LAI are given by the following equation:

4 $${\rm{LAI}}-\delta {{\rm{LAI}}}_{{\rm{Lower}}}\le {\rm{LAI}}\le {\rm{LAI}}+\delta {{\rm{LAI}}}_{{\rm{Upper}}}$$

where

4a $$\delta {{\rm{LAI}}}_{{\rm{Upper}}}=\frac{\sqrt{\sum ({{\rm{\delta }}}_{{{\rm{Upper}}}_{i}}^{2})}}{\mathrm{plot}\,\,{\rm{area}}}$$

4b $$\delta {{\rm{LAI}}}_{{\rm{Lower}}}=\frac{\sqrt{\sum ({{\rm{\delta }}}_{{{\rm{Lower}}}_{i}}^{2})}}{\mathrm{plot}\,\,{\rm{area}}},$$

and ${{\rm{\delta }}}_{{{\rm{Upper}}}_{i}}$ and ${{\rm{\delta }}}_{{{\rm{Lower}}}_{{i}}}$ are the errors of the tree LA of individual *i* calculated using equation (3). Error propagations for estimates of ΔLAI and Δ*B*_STEM_ were conducted in the same way. See S1–3 and S1–4 in Supplementary Information S1 for details.

### Meteorological data

We used the meteorological data recorded at the Automated Meteorological Data Acquisition System (AMeDAS) station at Inabu (35°12.7′N, 137°30.4′E, 505 m a.s.l.), located 5.4 km west of the study plot. At this station, the mean annual air temperature, mean monthly air temperature of the coldest month (January) and the warmest month (August), and mean annual precipitation were 11.1 °C, −0.2 °C and 22.9 °C, and 1901 mm, respectively, over the period of 1979 to 1996. There is a rainy season between early June and late July each year in the Tokai district, which includes the Inabu sites^59^. Precipitation and temperature records were available for the Inabu AMeDAS station from May and December 1978, respectively, which did not cover the entire range of our study period (1977–1996). To extrapolate the missing data, we used meteorological data recorded at the former Iida Weather Station (35°30.8′N, 137°50.0′E, 482 m a.s.l.), which was located 42 km northeast of our study site. Despite the distance, the correlation between Inabu and Iida for monthly mean air temperature was high (*R*^2^ = 0.996, residual SE = 0.50 °C mon^−1^; see Supplementary Information S2). By contrast, the correlation for the monthly precipitation was lower (*R*^2^ = 0.780, residual SE = 48.6 mm mon^−1^; see Supplementary Information S2). Because of the large SE value, we only used the data for the period of 1979 to 1996 from the Inabu AMeDAS station for analyses involving precipitation, and did not include estimates of monthly precipitation in 1977 and 1978 at the Inabu station based on Iida Weather Station data.

We estimated monthly potential evaporation between 1980 and 1996 from available data, using the methods of Xu *et al*.^60^, in which potential evaporation is defined as the evaporation expected from a continuously saturated surface. For details of these calculations, see Supplementary Information S3.

### Analyses using meteorological data

Previous studies have shown that not only the annual value of a meteorological factor but also that of specific months or seasons in a year can affect trees and forests^61–63^. Hence, preliminary analyses were carried out to identify the months in which temperature, precipitation, and potential evaporation data were most strongly correlated with the response variables representing stand properties (e.g., *B*_STEM_, LAI, and their rates of change). After trial-and-error testing, the sum of the monthly precipitation in May, June, and July of each year (*P*_MJJ_, mm), the average of the monthly mean air temperatures in July and August of each year (*T*_JA_, °C), and the monthly potential evaporation of July (*E*_July_, mm) were used. The minimum and the maximum values of *T*_JA_ were 20.3 °C (26-years stand age) and 24.2 °C (38-years stand age), respectively, with an average of 22.5 °C (21–40 years, including the estimated values from the Iida Weather Station data). The minimum and the maximum values of *P*_MJJ_ were 360 mm (38-years stand age) and 1004 mm (29-years stand age), respectively, with an average of 657 mm. There was no significant relationship between *T*_JA1y_ and *P*_MJJ_ for the period 1979–1996 (23–40 years of stand age) (*R*^2^ = 0.000, *P* = 0.905, *F*_(1,17)_ = 0.015).

## Electronic supplementary material

Supplementary Information
