# Supplementary material for: Interannual variability of leaf area index of an evergreen conifer stand was affected by carry-over effects from recent climate conditions
Source: Sci Rep. 2018 Sep 11;8:13590. doi: 10.1038/s41598-018-31672-3 (PMC6133949; doi:10.1038/s41598-018-31672-3)
Supplement: Supplementary file 1 — Supplementary Information [file 41598_2018_31672_MOESM1_ESM.docx]

**Supplementary Information for:**

**Interannual variability of leaf area index of an evergreen conifer stand was affected by carry-over effects from recent climate conditions**

Akihiro Sumida ^1,*^, Tsutomu Watanabe ^1^, and Tomiyasu Miyaura ^2^

^1^ Institute of Low Temperature Science, Hokkaido University, N19W8, Sapporo 060-0819, Japan;

^2^ Faculty of Science and Technology, Ryukoku University, Seta Oe-cho, Otsu, Shiga 520-2194, Japan

^*^ Corresponding author: asumida@lowtem.hokudai.ac.jp

**S1. Allometric equations, definition of errors, and error propagation for stand-level estimates**

**S1-1. Derivation of allometry for estimating tree leaf area.** The allometric relationship between the stem diameter at the crown base (*D*_CB_, cm) and tree leaf area (LA, m^2^) was determined as follows: We pooled the tree inventory data from three even-aged stands, Inabu (our study site), Dando (*ca.* 10-km south of Inabu), and Hourai (*ca.* 25-km south of Inabu) in the HYO Data. Forty-seven trees were represented in the pooled data. The ranges of *D*_CB_ and tree age were 2.3–22.3 cm and 16–59 years, respectively, which almost covered the ranges of *D*_CB_ (1.3–15.2 cm) and tree age (21–40 years) in the 20-year dataset in our study. After the values of *D*_CB_ and LA from the pooled data were natural-log transformed, regression coefficients were computed using an ordinary maximum likelihood method, and the following equation was derived:

ln(LA)＝−2.258＋2.460×ln(*D*_CB_), (S1)

where *R*^2^=0.942, *F*=743 on 45 *df*, *n*=47, and *P*<0.001. The correction factor for log transformation, (CF_LA_＝1.051 in equation (1) in the main text) was calculated as follows:

CF_LA_=exp(*s*^2^/2),

where *s* is the standard error of regression of equation (S1). By adding the correction factor to equation (S1), we obtained the following equation:

ln(LA)＝ln(1.051) −2.258＋2.460×ln(*D*_CB_), (S2)

which is identical to the natural-log transformed form of equation (1) in the main text. The allometric relationship on a log-log plane is illustrated in Fig. S1a, and this relationship is shown on a plane with a non-logarithmic vertical axis in Fig. S1b.


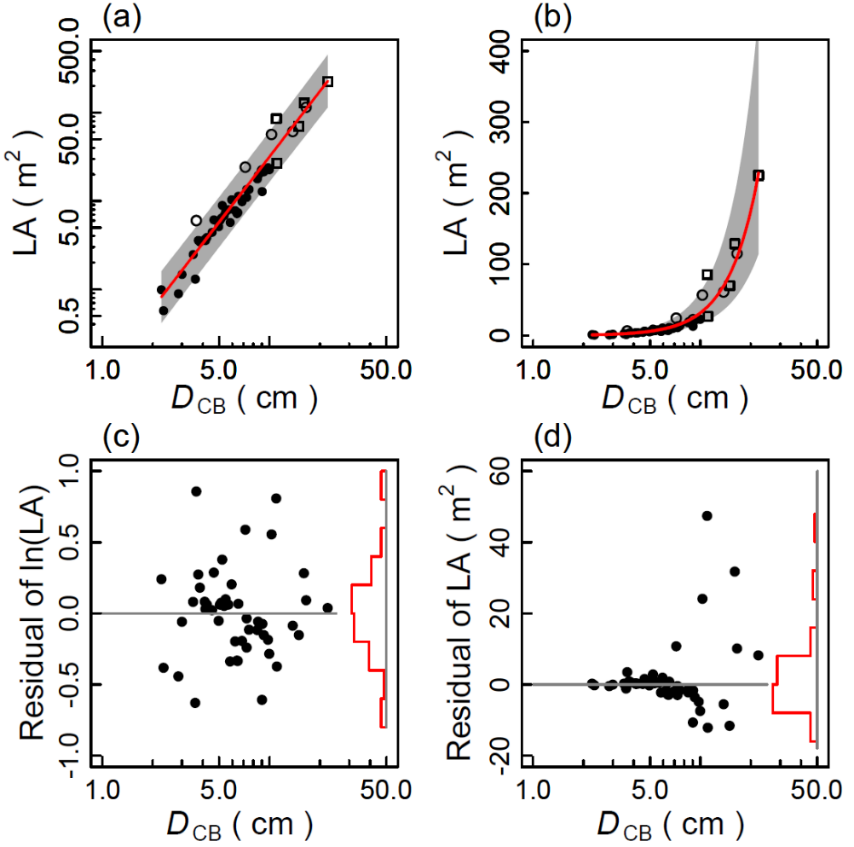


**Figure S1.** Pipe model allometry between stem diameter at crown base (*D*_CB_) and tree leaf area (LA). (a), Allometric line (equations (1) and (S2)) and 95% prediction intervals (gray area) on log-log plane; (b), allometric curve of (a) drawn on a plane with non-log vertical axis; (c), residuals expressed as ln(LA) of allometric relationship in (a); (d), residuals of (c) converted into non-log values of LA. In (a) and (b), open squares, open circles, and closed circles indicate data from Hourai, Dando, and Inabu sites, respectively. In (c) and (d), relative frequencies of residuals are shown.

As shown in Fig. S1c, the residuals of ln(LA) were normally distributed, suggesting that errors of the regression were randomly distributed around estimates. However, when these residuals of ln(LA) estimates were transformed into non-log values (Fig. S1d), the residuals of LA increased with increasing *D*_CB_, and the residuals were greater in the positive part than in the negative part (Fig. S1d). Considering this difference between the positive and the negative parts around an estimate, we defined the estimation errors of LA from allometry as described below.

**S1-2. Definition of errors of estimates calculated with allometric equation.** In Fig. S1, the upper and lower limits of the 95% prediction intervals (PIs) of ln(LA) were calculated^58^ as follows:

$$\mathrm{PI}\left( \ln(\mathrm{LA}_{\left（ {D_{\mathrm{CB}}}^{*} \right）}) \right)=\ln\left( \mathrm{LA}_{\left（ {D_{\mathrm{CB}}}^{*} \right）} \right)\pm t_{\left( \alpha/2,n-2 \right)}\times X, （S3）$$

where $\mathrm{LA}_{\left（ {D_{\mathrm{CB}}}^{*} \right）}$ indicates the predicted value of LA when $D_{\mathrm{CB}}$=${D_{\mathrm{CB}}}^{*}$, *n* is the sample size, $t_{(\alpha/2, n-2)}$ is the *t* value at a significance level *α* (=0.05), and *X* is given by the following equation^57^:

$X=s\times\sqrt{1+\frac{1}{n}+\frac{{({\ln(D_{\mathrm{CB}}}^{*})-\bar{\ln\left( D_{\mathrm{CB}} \right)} )}^{2}}{\sum\left( {\ln\left( D_{\mathrm{CB}} \right)}_{i}-\bar{\ln\left( D_{\mathrm{CB}} \right)} \right)^{2}}}$ , (S4)

where *s* is the residual SE of regression, and $\bar{\ln(D_{CB})}$ is the mean value of $\ln(D_{CB})$. In Fig. S1b, the 95% PI of a non-log value of $\mathrm{LA}_{\left（ {D_{\mathrm{CB}}}^{*} \right）}$ is represented by taking the exponential form of the right-hand side of equation (S3), as follows:

$${PI(LA}_{\left（ {D_{\mathrm{CB}}}^{*} \right）})=\exp\left\{ (ln(\mathrm{LA}_{\left（ {D_{\mathrm{CB}}}^{*} \right）})\pm t_{\left( \alpha/2, n-2 \right)}\times X \right\}, (S5)$$

where ${PI(LA}_{\left（ {D_{\mathrm{CB}}}^{*} \right）})$ is the PI of $\mathrm{LA}_{\left（ {D_{\mathrm{CB}}}^{*} \right）}$ on a plane with a non-log vertical axis, which corresponds to the $\mathrm{PI}\left( \ln(\mathrm{LA}_{\left（ {D_{\mathrm{CB}}}^{*} \right）}) \right)$ on a log-log plane, and *X* is given by equation (S4).

As stated in the main text, the error of $\mathrm{LA}_{\left（ {D_{\mathrm{CB}}}^{*} \right）}$ was defined by using the 95% PIs (equation (S5)) as follows:

$\delta_{\mathrm{Upper}\left（ {D_{\mathrm{CB}}}^{*} \right）}=\mathrm{PI}_{\mathrm{Upper}\left（ {D_{\mathrm{CB}}}^{*} \right）}-\mathrm{LA}_{\left（ {D_{\mathrm{CB}}}^{*} \right）}$ (S6a) (=equation (3a))

$\delta_{\mathrm{Lower}{{(D}_{\mathrm{CB}}}^{*})}={\mathrm{LA}_{\left（ {D_{\mathrm{CB}}}^{*} \right）}-PI}_{\mathrm{Lower}\left（ {D_{\mathrm{CB}}}^{*} \right）}$ (S6b) (=equation (3b))

where $\delta_{\mathrm{Upper}\left（ {D_{\mathrm{CB}}}^{*} \right）}$ and $\delta_{\mathrm{Lower}{{(D}_{\mathrm{CB}}}^{*})}$ indicate the errors defined by the upper and lower limits of the 95% PIs, respectively (equation (S5)). Then, the upper and lower error intervals of $\mathrm{LA}_{\left（ {D_{\mathrm{CB}}}^{*} \right）}$ were calculated by substituting the PIs (equation (S5)) into equation (S6) as follows:

$$\delta_{\mathrm{Upper}\left（ {D_{\mathrm{CB}}}^{*} \right）}=\exp\left\{ (ln(\mathrm{LA}_{\left（ {D_{\mathrm{CB}}}^{*} \right）})+t_{\left( 0.05/2,n-2 \right)}\times X \right\}-\mathrm{LA}_{\left（ {D_{\mathrm{CB}}}^{*} \right）} (S7a)$$

$$\delta_{\mathrm{Lower}{{(D}_{\mathrm{CB}}}^{*})}=\mathrm{LA}_{\left（ {D_{\mathrm{CB}}}^{*} \right）}-exp\left\{ (ln(\mathrm{LA}_{\left（ {D_{\mathrm{CB}}}^{*} \right）})-t_{\left( 0.05/2,n-2 \right)}\times X \right\} (S7b)$$

Mathematically, $\delta_{\mathrm{Upper}\left（ {D_{\mathrm{CB}}}^{*} \right）}$ is ≥ $\delta_{\mathrm{Lower}{{(D}_{\mathrm{CB}}}^{*})}$, which corresponds to the fact that the absolute values of errors are greater in the upper part than in the lower part around the regression line in Fig. S1b.

Note that the error defined by the 95% PIs (equation (S7)) corresponds to an estimate of ±2σ (=2×s.d.). This definition of error is conservative in that errors are often (implicitly) defined by estimating ±1σ (=1×s.d.), or by 68.3% PIs (Taylor 1997). Thus, in addition to the error defined using 95% PIs (equation (S7)), we also defined the error corresponding to estimate±1σ, $\sigma_{\mathrm{Upper}\left（ {D_{\mathrm{CB}}}^{*} \right）}$ and $\sigma_{\mathrm{Lower}{{(D}_{\mathrm{CB}}}^{*})}$. This was calculated by replacing the significance level of the *t*-value in equation (S7) with *α*=0.317 (=1−0.683), as follows:

$$\sigma_{\mathrm{Upper}\left（ {D_{\mathrm{CB}}}^{*} \right）}=\exp\left\{ (ln(\mathrm{LA}_{\left（ {D_{\mathrm{CB}}}^{*} \right）})+t_{\left( 0.317/2 ,n -2 \right)}\times X \right\}-\mathrm{LA}_{\left（ {D_{\mathrm{CB}}}^{*} \right）} (S8a)$$

$$\sigma_{\mathrm{Lower}{{(D}_{\mathrm{CB}}}^{*})}=\mathrm{LA}_{\left（ {D_{\mathrm{CB}}}^{*} \right）}-\exp\left\{ (ln(\mathrm{LA}_{\left（ {D_{\mathrm{CB}}}^{*} \right）})-t_{\left( 0.317/2 ,n -2 \right)}\times X \right\} (S8b)$$

where $\sigma_{\mathrm{Upper}\left（ {D_{\mathrm{CB}}}^{*} \right）}$ and $\sigma_{\mathrm{Lower}{{(D}_{\mathrm{CB}}}^{*})}$ correspond to 1σ above and below the $\mathrm{LA}_{\left（ {D_{\mathrm{CB}}}^{*} \right）}$, respectively.

**S1-3. Error propagation for LAI estimates.** After the errors were calculated for all estimated tree LAs, these errors were propagated to an estimate of LAI (the sum of tree LAs divided by the plot area). According to the rule of error propagation (Taylor 1997), the upper and the lower limits of LAI were calculated using the definition of error (equation (S7)), as follows.

$\delta\mathrm{LAI}_{\mathrm{Upper}}=\frac{\sqrt{\sum{({\delta_{\mathrm{Upper}}}_{i}}^{2})}}{plot area} (S9a)$ (=equation (4a))

$\delta\mathrm{LAI}_{\mathrm{Lower}}=\frac{\sqrt{\sum{({\delta_{\mathrm{Lower}}}_{i}}^{2})}}{plot area} (S9b)$ (=equation (4a))

Consequently, the error boundaries of LAI based on the 95% PIs of tree LA could be written as follows:

$$\mathrm{LAI}-\delta\mathrm{LAI}_{\mathrm{Lower}} \leq\mathrm{LAI}\leq\mathrm{LAI}+\delta\mathrm{LAI}_{\mathrm{Upper}} . (S10)$$

**S1-4. Error propagation for ΔLAI estimates.** Care must be taken when propagating errors for a ΔLAI estimate, because it is the sum of the differences between LAs in two years, LA(*t*)±δ(*t*) and LA(*t*−1)±δ(*t*−1). From equation (3) in the main text, we first rewrote the error intervals of LA at years *t* and (*t−*1) for a tree *i* as shown below:

$${\mathrm{LA}\left( t \right)}_{i}-{{\delta\left( t \right)}_{i}}_{\mathrm{Lower}} \leq{\mathrm{LA}\left( t \right)}_{i}\leq{\mathrm{LA}\left( t \right)}_{i}+{{\delta\left( t \right)}_{i}}_{\mathrm{Upper}} , (S11a)$$

$${\mathrm{LA}\left( t-1 \right)}_{i}-{{\delta\left( t-1 \right)}_{i}}_{\mathrm{Lower}} \leq{\mathrm{LA}\left( t-1 \right)}_{i}\leq{\mathrm{LA}\left( t-1 \right)}_{i}+{{\delta\left( t-1 \right)}_{i}}_{\mathrm{Upper}} , (S11b)$$

where ${{\delta\left( t \right)}_{i}}_{\mathrm{Lower}}$and ${{\delta\left( t \right)}_{i}}_{\mathrm{Upper}}$ indicate absolute values of the lower and upper errors, respectively, defined by equation (S7) for year *t*. Multiplying all terms of expression in (S11b) by −1 resulted in:

$$-{\mathrm{LA}\left( t-1 \right)}_{i}-{{\delta\left( t-1 \right)}_{i}}_{\mathrm{Upper}}\leq-\mathrm{LA}_{i}\left( t-1 \right)\leq-{\mathrm{LA}\left( t-1 \right)}_{i}+{{\delta\left( t-1 \right)}_{i}}_{\mathrm{Lower}} . (S11c)$$

Then, adding each side of (S11a) to (S11c), we obtained the following error boundaries of ΔLA of a tree *i*:

$${\Delta LA}_{i}-\left( {{\delta\left( t \right)}_{i}}_{\mathrm{Lower}}+{{\delta\left( t-1 \right)}_{i}}_{\mathrm{Upper}} \right)\leq{\Delta LA}_{i}\leq{\Delta LA}_{i}+\left( {{\delta\left( t \right)}_{i}}_{\mathrm{Upper}}+{{\delta\left( t-1 \right)}_{i}}_{\mathrm{Lower}} \right), (S12)$$

where ${\Delta LA}_{i}=\mathrm{LA}_{i}\left( t \right)-\mathrm{LA}_{i}\left( t-1 \right)$.

The parentheses in expression (S12) indicate the upper and lower error intervals for a ΔLA*_i_*, which we expressed using ${\delta{\Delta LA}_{i}}_{\mathrm{Upper}}$ and ${\delta{\Delta LA}_{i}}_{\mathrm{Lower}}$ as follows:

${\delta{\Delta LA}_{i}}_{\mathrm{Upper}}={{\delta(t)}_{i}}_{\mathrm{Upper}}+{{\delta\left( t-1 \right)}_{i}}_{\mathrm{Lower}}$ (S13a)

${\delta{\Delta LA}_{i}}_{\mathrm{Lower}}={{\delta(t)}_{i}}_{\mathrm{Lower}}+{{\delta\left( t-1 \right)}_{i}}_{\mathrm{Upper}}$ (S13b).

These errors of individual ΔLAs were used to calculate the upper and lower limits of ΔLAI. Here, we note that ${{\delta(t)}_{i}}_{\mathrm{Upper}}$ and ${{\delta\left( t-1 \right)}_{i}}_{\mathrm{Lower}}$, and ${{\delta(t)}_{i}}_{\mathrm{Lower}}$ and ${{\delta\left( t-1 \right)}_{i}}_{\mathrm{Upper}}$, were values for the same tree. Hence, it is likely that they were correlated. In this case, the upper and lower errors of ΔLAI should be calculated using the rule of error propagation considering covariance among errors (Taylor 1997), as follows:

$$\delta{\Delta LAI}_{\mathrm{Upper}}=\frac{\sqrt{\sum{({\delta{\Delta LA}_{i}}_{\mathrm{Upper}}}^{2})}}{plot area} (S14a)$$

$$\delta{\Delta LAI}_{\mathrm{Lower}}=\frac{\sqrt{\sum{({\delta{\Delta LA}_{i}}_{\mathrm{Lower}}}^{2})}}{plot area} , (S14b)$$

where ${\delta{\Delta LA}_{i}}_{\mathrm{Upper}}$ and ${\delta{\Delta LA}_{i}}_{\mathrm{Lower}}$ are given by equation (S13).

The errors of LAI and ΔLAI defined with 1×σ=1×s.d. (equation (S8)) were calculated in the same ways.

**S1-5. Derivation of allometry for estimating stem dry weight, stem biomass, stem growth rate, and their errors**

The allometric relationship between the stem volume *V*_S_ (m^3^) and stem dry weight *W*_S_ (kg) was determined using the same procedure as that used to determine tree LA. That is, we pooled the tree data from three stands, Inabu, Dando, and Hourai in the HYO Data to derive the allometry. The range of *V*_S_ (0.00276–0.783 m^3^) in the HYO data almost covered that of our study (0.00161–0.277 m^3^). A regression calculation was carried out using natural-log transformed values, and the following equation was obtained.

ln(*W*_S_)＝5.905＋0.975×ln(*V*_S_), (S15)

where *R*^2^=0.996, *F*=18000 on 45 *df*, *n*=47, and *P*=0.001. The correction factor for log transformation was: CF_WS_＝1.002. By adding this correction factor to equation (S15), we obtained the following:

ln(*W*_S_)＝ln(1.002) +5.905＋0.975×ln(*V*_S_), (S16)

which is identical to the natural-log transformed form of equation (2) in the main text. The upper and lower limits of 95% PI were calculated as in equation (S6), and the errors for the estimates of *W*_S_ corresponding to 2σ and 1σ were defined and calculated as in equations (S7) and (S8), respectively. The allometric relationship and its 95% PIs are shown with their residuals in Fig. S2. Because of the very high *R*^2^ value for equation S15, the vertical ranges of 95% PIs were very narrow, as shown in Fig. S2a.


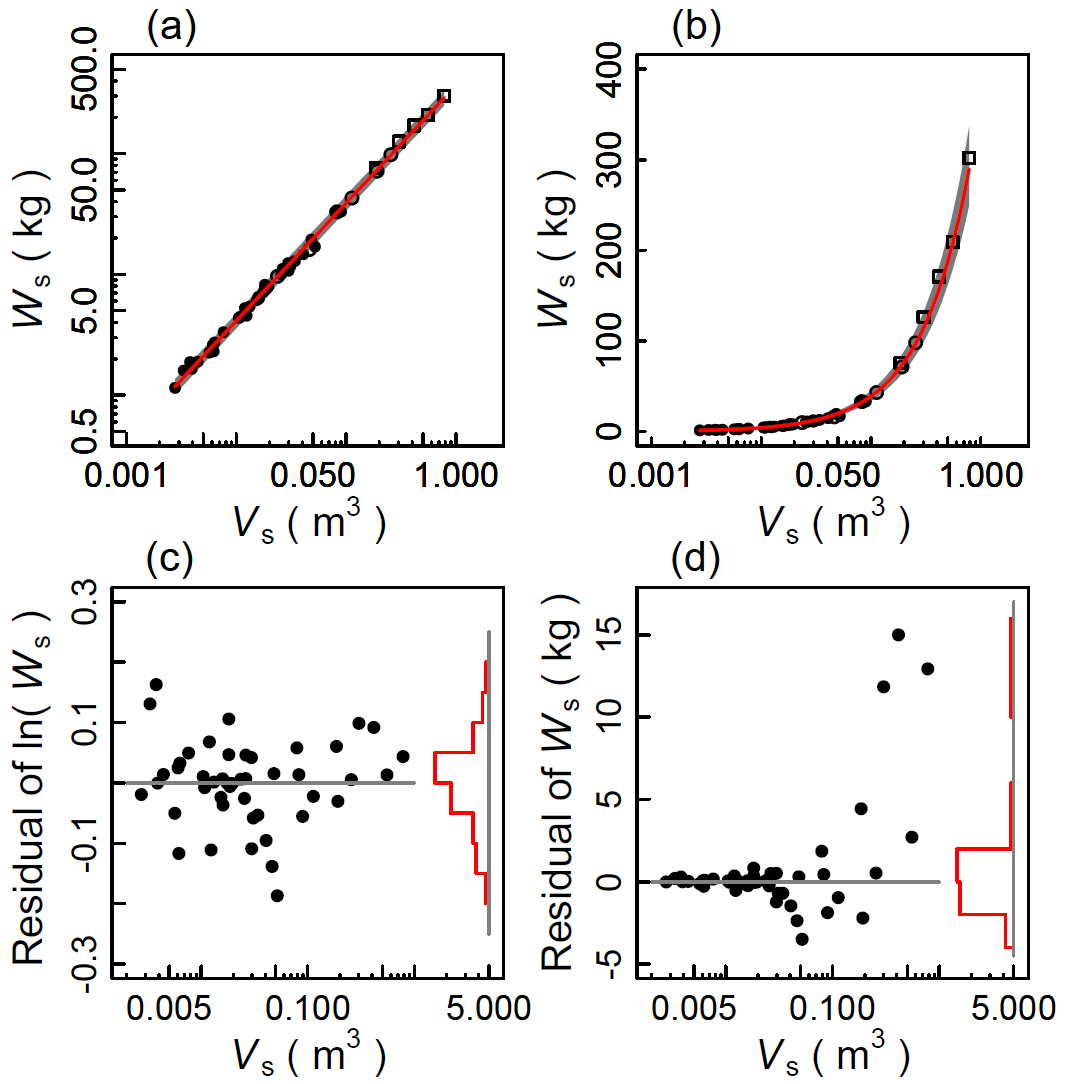
 The errors of the stand stem biomass *B*_STEM_ (kg m^−2^) and its growth rate (Δ*B*_STEM_, kg m^−2^ year^−1^) were defined and calculated in the same way as those for LAI and ΔLAI.

**Figure S2.** Allometric relationship between stem volume (*V*_S_) and stem dry weight (*W*_S_). (a) Allometric line (equations (2) and (S16)) on a log-log plane; 95% PIs (grey area) are shown, but are almost hidden by regression line due to their narrow vertical width; (b), allometric curve of (a) drawn on plane with non-log vertical axis; (c), residuals expressed as ln(*W*_S_) of allometric relationship in (a); (d), residuals of (c) converted into non-log values of *W*_S_. In (a) and (b), open squares, open circles, and closed circles are data from Hourai, Dando, and Inabu sites, respectively. In (c) and (d), relative frequencies of residuals are shown. **S2 Estimation of monthly mean air temperature and precipitation for missing years**

­­

**Figure S3.** Relationships between Iida and Inabu of (a) monthly mean air temperatures (*T*_Iida_ and *T*_Inabu_; °C mon^−1^) and (b) monthly precipitations (*P*_Iida_ and *P*_Inabu_; mm mon^−1^) between 1979 and 1997.

The regression for monthly mean temperature was given by:

*T*_Inabu_ = −1.11 + 0.98×*T*_Iida_ , (S17)

where *R*^2^=0.996, residual SE = 0.496 °C mon^−1^, *F*=62200 on 226 *df*, *P*=0.000.

The regression for monthly precipitation was given by:

*P*_Inabu_ = 16.6 + 1.09×*P*_Iida_ , (S18)

where *R*^2^=0.780, residual SE = 48.6 mm mon^−1^, *F*=805 on 226 *df, P*=0.000.

**S3. Monthly potential evaporation**


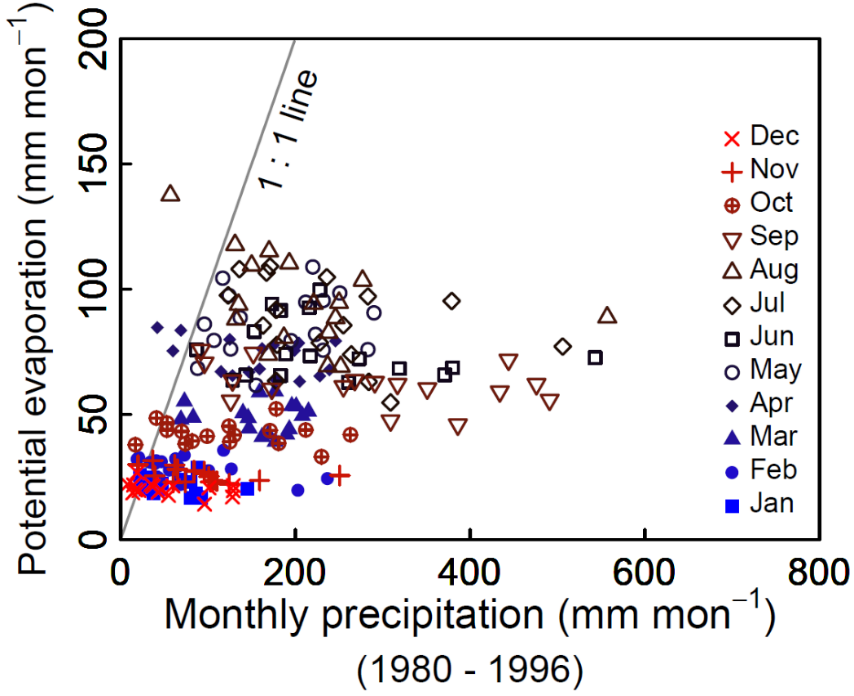
Monthly potential evaporation was estimated according to the method of Xu *et al*.^60^, where potential evaporation is defined as the evaporation expected from a continuously saturated surface (Xu *et al*.^60^). The data used for these estimates were air temperature, wind speed, precipitation, and daily hours of sunshine recorded at the AMeDAS station at Inabu, and air temperature, air pressure, and relative humidity recorded at the Iida Weather Station (for information on these stations, see Materials and Methods section in the main text). Monthly potential evaporation between 1980 and 1996 (Fig. S4) was estimated using the available data. As shown in the figure, monthly potential evaporation in the study plot seldom exceeded monthly precipitation, even in summer.

**Figure S4.** Relationship between monthly potential evaporation and monthly precipitation estimated for study plot between January 1980 and December 1996.

**S4. Effects of number of years for calculating moving averages of mean summer temperature.**

**Figure S5.** Relationships between LAI and *T*_JA_*_N_*_y_ (moving averages of *T*_JA_'s (mean air temperature of July and August) of past *N*_y_ years). Tested values of *N*_y_ (number of past years used to calculate moving average) ranged from 1 to 10, where *N*_y_ = 1 represents current year. Left-hand panel, chronological changes in LAI and *T*_JA_*_N_*_y_. Right-hand panel, relationships between LAI and *T*_JA_*_N_*_y_ with *R*^2^ and *P*-values of regression. Figures for *N*_y_ = 1 (current-year only) and *N*_y_ = 6 (six-year averages) are the same as Fig. 4e,f in main text. Data points are connected with straight lines in chronological order. Thick and thin vertical lines show propagated errors defined for 1σ and 2σ, respectively.


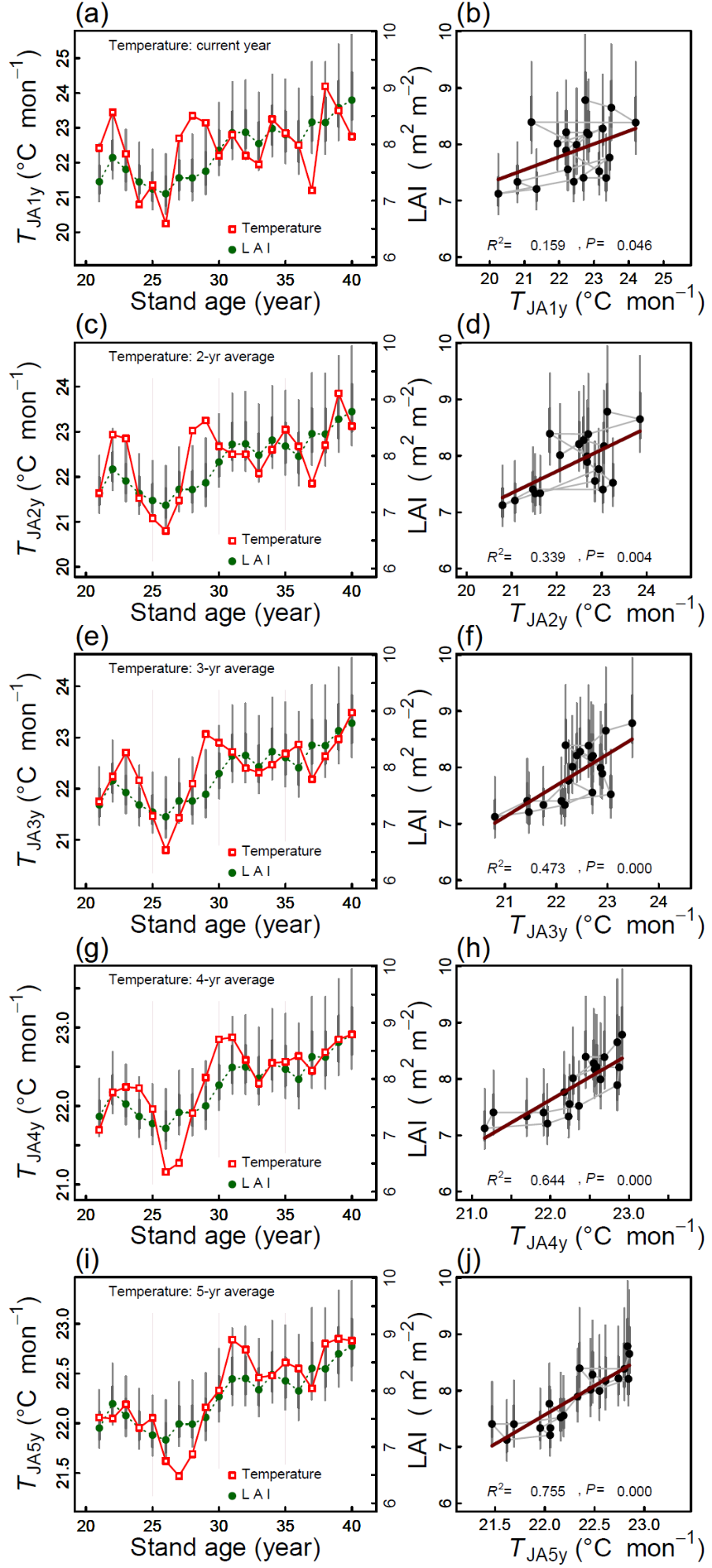


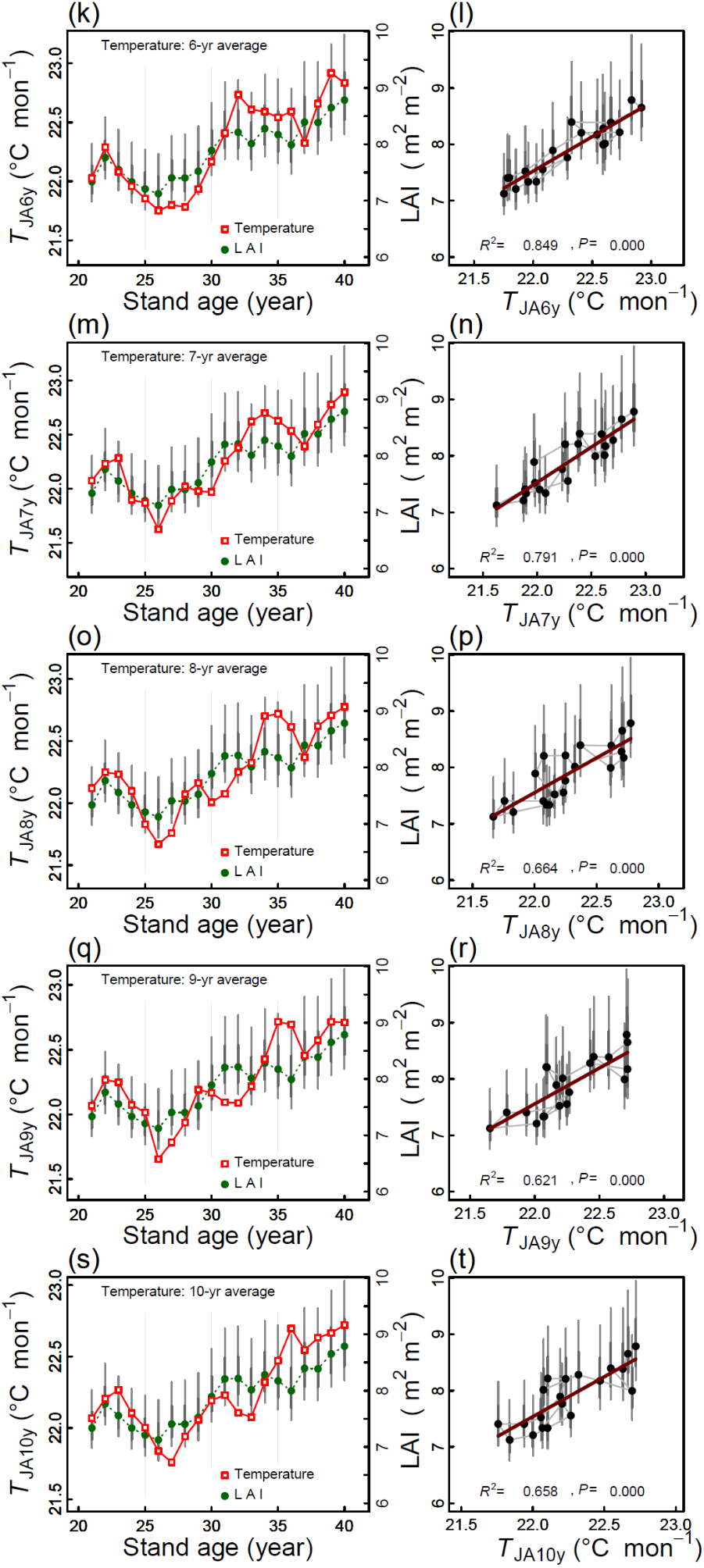

**S5. Distribution of the sum of tree LAs for each tree height class**

**Fig. S6**. Distribution of the sum of tree LAs (green bars) for the trees belonging to each tree height class in each year. The height of each pink bar indicates the tree LA sums for those trees in which tree LA decreased from that of the previous year, or for the trees with ∆LA<0, of which the share for the dying trees are shown by yellow bars. Thus, the pink portion excludes LAs of the dying trees. For the trees with ∆LA<0, the proportion of the sum of tree LAs to the stand LAI (rLAI_(∆LA<0)_) is indicated by the percentage in the upper-right corner of each plot. Purple bars are the sum of tree LAs for the trees that died before the following year. For these trees, the percentage of the stand LAI is indicated by the value in parentheses. There was a significant positive relationship between rLAI_(∆LA<0)_ and *R*_(∆LA<0)_ in Fig 4: rLAI_(∆LA<0)_=−0.121+0.773×*R*_(∆LA<0)_ , where *R*^2^=0.838, *F*=93.74 on 1 and 17 *df*, *P*=0.000.
